# Combination of Gonadal Dysgenesis and Monosomy X with a Novo Translocation (13,14)

**DOI:** 10.1155/2018/3796415

**Published:** 2018-12-17

**Authors:** Hanane Latrech, Houssein Madar, Ahmed Gaouzi

**Affiliations:** ^1^Department of Endocrinology, Mohammed VI Hospital, Medical School, University Mohamed First, 60 000 Oujda, Morocco; ^2^Department of Endocrinology Paediatrics, Medical School, Mohammed V University, Hôpital d'Enfants, 10 000 Rabat, Morocco

## Abstract

Turner syndrome is a common sex chromosome disorder characterized by complete or partial absence of an X chromosome. The spectrum of its clinical features and cytogenetics are various. We report new chromosomal formula revealed by DSD and associated with translocation (13,14). To our knowledge, this is the first case of 45X, t(13;14) de novo translocation as a variation of Turner syndrome in a patient with this clinical presentation.

## 1. Introduction

Turner syndrome is a common genetic disorder due to complete or partial absence of an X chromosome that affects 1 in every 2000 girls [1–3]. The phenotypic features are various and associate usually with short stature, dysmorphic syndrome, and gonadal dysgenesis. Webbed neck, lymphedema, cubitus valgus, cardiovascular malformations, bones and thyroid disorders, and renal and liver dysfunctions may also be observed. Cytogenetically, the Turner syndrome is characterized by sex chromosome monosomy (45X) in 50-60% of the cases. In addition to this anomaly, structural and numerical mosaicism are seen rather frequently [1, 3, 4]. We report a case of gonadal dysgenesis revealed monosomy X associated with a novo translocation (13,14). This association has not been reported previously in the literature.

## 2. Observation

**Figure 1:**
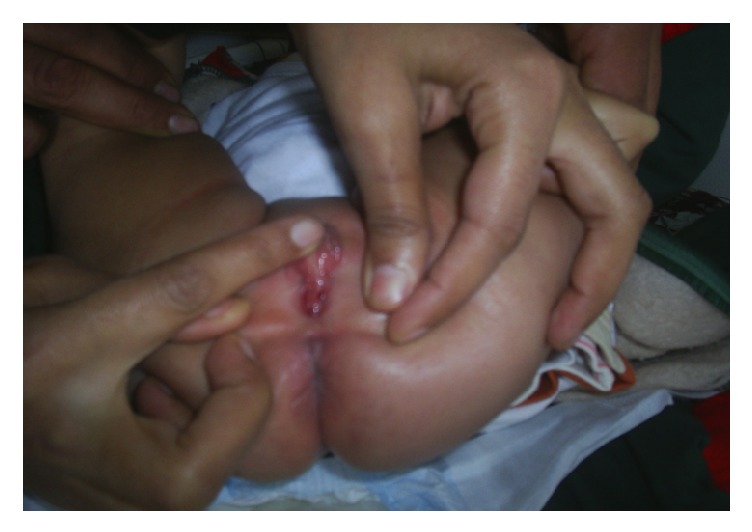
Appearance of the external genitalia.

**Figure 2:**
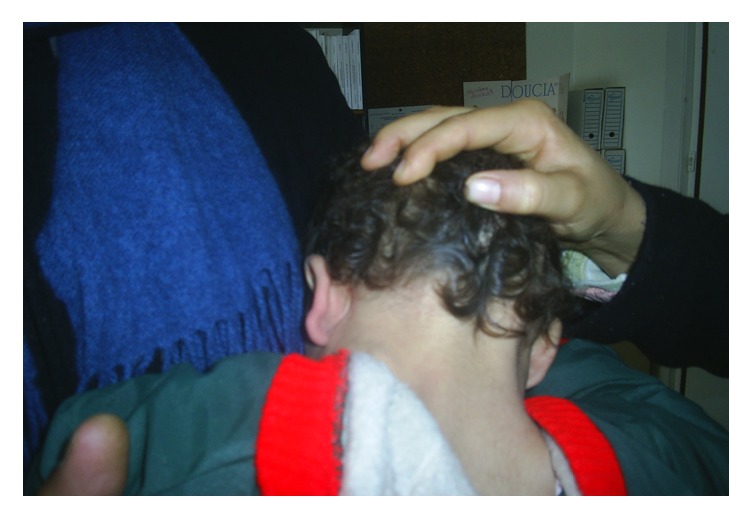
Low hairline and low dysmorphic auricles.

A 3-month-old child assigned as a girl at birth was referred to our institution for disorder of sex development. There was no family history of consanguinity or similar case. The patient was born at 41 weeks of gestational age after uncomplicated pregnancy and delivery with normal birth weight and height and normal subsequent growth. Physical examination revealed a Prader 3 external genitalia (genital bourgeon 2 × 1 cm with presence of a single vulvar orifice, a female labioscrotal swelling) (Figure 1) and two palpables gonads in inguinal canal suggestive of gonadal tissue. There were a low hairline and low dysmorphic auricles with the longitudinal axis in an oblique position (Figure 2) without webbed neck or hands and/or feet lymphedema or other somatic abnormalities. An initial endocrine evaluation revealed a normal cortisol 8am and 17 OH progesterone and a level of baseline serum testosterone at 1.04 ng/ml, an FSH at 5.33 mIU/L, and a LH at 1.29 mIU/L. The abdominal-pelvic ultrasound showed uterus with the presence of two inguinal testes. Chromosome studies on blood lymphocytes were studied by conventional cytogenetics, with an analysis of 50 mitosis, which showed monosomy of chromosome X (45X) with the presence of a subpopulation with a derived of the chromosome 14 probably by (13,14) translocation. This translocation has occurred de novo because parent's karyotype was normal.

**Figure 3:**
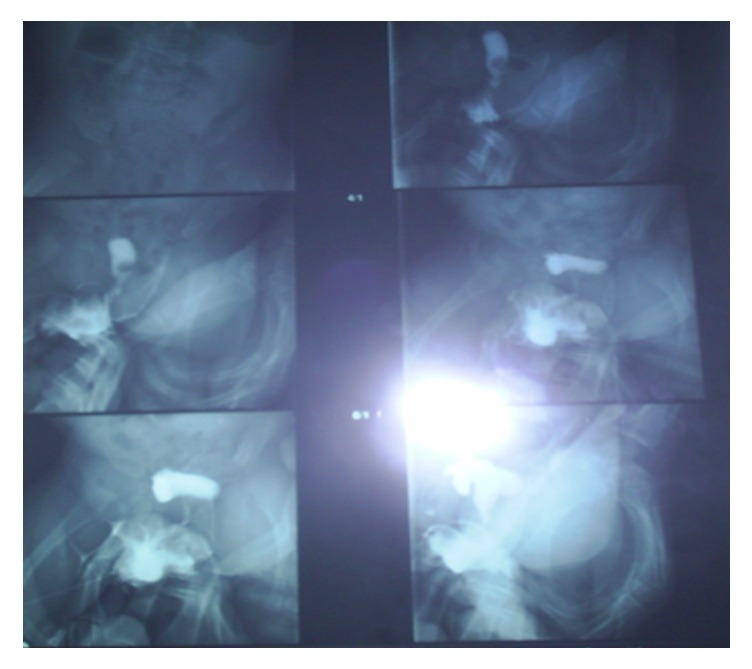
Genitography: female urethra, vaginal and uterine cavity, and fallopian tubes.

In the search of the SRY gene during polymerase chain reaction (PCR), the presence of SRY gene was not found. The FISH screening (Kreatech: SE Y (DYZ, green, pKBI-20024G) in the peripheral lymphocytes, analyzed in 20 nuclei, did not find the specific green spot of Y chromosome. The genitography found a female urethra, a vaginal and uterine cavity, and fallopian tubes (Figure 3). Laparoscopic exploration showed a vagina, uterus, and fallopian tubes with the presence of two gonads. Histological study of gonadal biopsies revealed testicular tissue with the presence of regular size seminiferous tubules containing Sertoli cells without the presence of germ or Leydig cell. A cardiovascular, renal, and auditory systems examination were normal. In a staff, bringing together endocrinologists and pediatric surgeons, the majority opinion was to maintain the female gender of the child after parental consent and achieve a feminizing genitoplasty (vaginoplasty and clitoridoplasty) with bilateral gonadectomy.

## 3. Discussion

Turner syndrome is one of the most common types of aneuploidy. 50% of patients with Turner syndrome have nonmosaic monosomy X (45X0) [1, 5]. Abnormalities of number or structure of the sex chromosome lead to alterations in gonadal development. Our case presents a gonadal dysgenesis associating with the presence of Mullerian ducts (uterine and vaginal cavities, fallopian tubes) and palpable gonad in inguinal position and low hairline as the only clinical phenotypic finding in Turner syndrome. Biopsy of the gonads revealed testicular tissue whose endocrine function, evaluated by level of baseline serum testosterone, appears to be altered. In this situation, the detection of mosaicism is essential. The classical cytogenetics that requires a large number of cells detects only a small percentage of mosaicism. Hence there is the need to use the fluorescence in situ hybridization (FISH) and the polymerase chain reaction (PCR) which can improve greatly the detection of low-frequency cell line and possible structural abnormalities. Peripheral blood lymphocytes are the material of choice for cytogenetic analysis on patients with monosomy X [3, 6–8]. In our case, the detection of Y material by PCR and FISH was negative. The chromosome studies carried out on peripheral lymphocytes do not always reflect the proportion of cell lines in the gonads. The detection of Y chromosome material in a gonadal tissue could be necessary given the risk of malignant transformation [3, 6]. This complementary research in the gonadal tissue or another tissue (buccal mucosa) could not be performed in our case. Of all the ways, our multidisciplinary staff had decided with parental consent to maintain the female gender of the child and achieve a feminizing genitoplasty with bilateral gonadectomy. Regular monitoring of other specific characteristics finding of Turner syndrome was proposed. In addition to the difficulty of the gonadal dysgenesis management in the presence of monosomy X, our case illustrates an association with autosomal translocation. This translocation was considered de novo as result of normality parent's karyotype. Translocations can be balanced or unbalanced and it is admitted that a balanced translocation does not show any phenotypic anomalies as in the case of our patient [9, 10]. X autosomal anomalies had been reported in the Turner syndrome [11, 12]. Coexistence of monosomy X with autosomal translocations is rarely described in the literature with only few cases reported [10, 13–18].

Robertsonian translocations (ROBs) are whole arm rearrangements involving the acrocentric 13-15 and 21-22 chromosomes and carriers are at increased risk for aneuploidy and thus uniparental disomy (UPD). In our case, we had not tested the patient for UPD 13 or 14 because the patient's phenotype does not seem suspicious for UPD 13 or 14.

The first case of Turner's syndrome with familial translocation was reported in 1979 in a patient with a number of clinical signs of Turner's syndrome with karyotype 45,X,t(1;2) (q32;q21) [13]. Ozkul reported a patient with Turner somatic features with a 45X,t(1;2) (q41;p11.2) karyotype [10]. Laszlo et al. reported a monosomy X with streak gonad syndrome associated with familial balanced translocation (13;14) [14]. In a cohort of 146 Serbian patients with Turner syndrome, a 12-year old girl with short stature, primary amenorrhea with autosomal translocation of chromosomes 12 and 14, that is, 45X/46XX, t(12,14), was found [15]. Cetin et al. reported an inherited reciprocal translocation and structural abnormality of the X chromosome (isochromosome Xq) in 16-year-old patient with Turner somatic features [16]. Our patient did not present at that time evident Turnerian phenotype. To our knowledge, this is the first case of 45X, t(13;14) de novo translocation as a variation of Turner syndrome in a patient with this clinical presentation including gonadal dysgenesis. This leads to raise the question of the relationship between the monosomy X and translocation and if balanced translocation might have influenced nondisjunction of the X chromosome. An interchromosomal effect does not appear in our case and this translocation seems to be an incidental finding without causal link.

## 4. Conclusion

Turner syndrome is one of the most common sex chromosome abnormalities. The spectrum of clinical manifestation and cytogenetics of Turner syndrome are various. In more than half of the cases, it is characterized by sex chromosome monosomy (45X). We report new chromosomal formula revealed by DSD and associated with translocation (13,14). To our knowledge, this is the first case of 45X, t(13;14) de novo translocation as a variation of Turner syndrome in a patient with this clinical presentation. To date, there seems to be no relationship between the two chromosomal abnormalities in the present case.
